# Structural Analysis of the UBA Domain of X-linked Inhibitor of Apoptosis Protein Reveals Different Surfaces for Ubiquitin-Binding and Self-Association

**DOI:** 10.1371/journal.pone.0028511

**Published:** 2011-12-15

**Authors:** Man Kit Tse, Sin Kam Hui, Yinhua Yang, Si-Tao Yin, Hong-Yu Hu, Bing Zou, Benjamin Chun Yu Wong, Kong Hung Sze

**Affiliations:** 1 Department of Microbiology and State Key Laboratory for Emerging Infectious Diseases, The University of Hong Kong, Hong Kong SAR, People's Republic of China; 2 Department of Chemistry and Open Laboratory of Chemical Biology of the Institute of Molecular Technology for Drug Discovery and Synthesis, The University of Hong Kong, Hong Kong SAR, People's Republic of China; 3 Department of Biochemistry, Centre for Protein Science and Crystallography, The Chinese University of Hong Kong, Shatin, Hong Kong SAR, People's Republic of China; 4 State Key Laboratory of Molecular Biology, Institute of Biochemistry and Cell Biology, Shanghai Institutes for Biological Sciences, Chinese Academy of Sciences, Shanghai, People's Republic of China; 5 Department of Medicine, The University of Hong Kong, Pokfulam Road, Hong Kong SAR, People's Republic of China; MRC National Institute for Medical Research, United Kingdom

## Abstract

**Background:**

Inhibitor of apoptosis proteins (IAPs) belong to a pivotal antiapoptotic protein family that plays a crucial role in tumorigenesis, cancer progression, chemoresistance and poor patient-survival. X-linked inhibitor of apoptosis protein (XIAP) is a prominent member of IAPs attracting intense research because it has been demonstrated to be a physiological inhibitor of caspases and apoptosis. Recently, an evolutionarily conserved ubiquitin-associated (UBA) domain was identified in XIAP and a number of RING domain-bearing IAPs. This has placed the IAPs in the group of ubiquitin binding proteins. Here, we explore the three-dimensional structure of the XIAP UBA domain (XIAP-UBA) and how it interacts with mono-ubiquitin and diubiquitin conjugates.

**Principal Findings:**

The solution structure of the XIAP-UBA domain was determined by NMR spectroscopy. XIAP-UBA adopts a typical UBA domain fold of three tightly packed α-helices but with an additional N-terminal 3_10_ helix. The XIAP-UBA binds mono-ubiquitin as well as Lys48-linked and linear-linked diubiquitins at low-micromolar affinities. NMR analysis of the XIAP-UBA–ubiquitin interaction reveals that it involves the classical hydrophobic patches surrounding Ile44 of ubiquitin and the conserved MGF/LV motif surfaces on XIAP-UBA. Furthermore, dimerization of XIAP-UBA was observed. Mapping of the self-association surface of XIAP-UBA reveals that the dimerization interface is formed by residues in the N-terminal 3_10_ helix, helix α1 and helix α2, separate from the ubiquitin-binding surface.

**Conclusion:**

Our results provide the first structural information of XIAP-UBA and map its interaction with mono-ubiquitin, Lys48-linked and linear-linked diubiquitins. The notion that XIAP-UBA uses different surfaces for ubiquitin-binding and self-association provides a plausible model to explain the reported selectivity of XIAP in binding polyubiquitin chains with different linkages.

## Introduction

The IAP (inhibitor of apoptosis) proteins are an evolutionarily conserved family of cell-death regulators, which can block apoptosis induced by diverse stimuli through direct interactions with a variety of inducers and effectors of apoptosis [1]–[3]. This places IAPs in a central position as inhibitors of death signals that proceed through a number of different pathways. Defects in apoptosis play an important role in the pathogenesis of a number of diseases such as cancer, neurodegenerative and auto-immune diseases. Indeed, IAPs are often overexpressed in tumors, and they have been implicated in tumor development and progression, chemoresistance and poor patient survival [4]. X-linked inhibitor of apoptosis protein (XIAP) is the most versatile inhibitor of caspases and apoptosis *in viv*o [5]. XIAP is also a cancer biomarker, and it is regarded as a promising target for the development of anticancer drugs.

XIAP contains three N-terminal zinc-binding baculovirus IAP repeat (BIR) domains that have been shown to inhibit the activities of caspase-3, caspase-7 and caspase-9 [6], [7] as well as to mediate the activation of NF-κB pathway via interaction with TAK1 [8]. XIAP also possesses a C-terminal really interesting new gene (RING) domain that functions as an E3 ubiquitin ligase. The RING domain of XIAP ubiquitinates a wide range of substrates, thereby, affecting a broad range of cellular activities beyond apoptotic suppression [9]. There is now compelling evidences that XIAP also has significant roles in cell division, morphogenesis, heavy metal homeostasis, NF-κB activation and MAP kinase signaling [9], [10]. The long stretch of sequence (∼100 residues) linking the BIR3 domain to the C-terminal RING domain was not known to contain globular structural elements or functional components until recently. Gyrd-Hansen *et al.* reported that they had identified an evolutionarily conserved ubiquitin-associated (UBA) domain within this region using sequence analysis and structure prediction algorithm [11]. They also reported that similar UBA domains also exist in other RING-bearing IAP members, including XIAP, cellular IAP -1 and -2 (cIAP-1 and -2) and IAP-like protein 2 (ILP-2) [11], [12]. The conjugation of ubiquitin to target proteins plays a crucial part in the formation of signaling networks. The ubiquitination is mediated through low-affinity, non-covalent interactions between ubiquitin and small ubiquitin-binding domains present in specialized proteins that are collectively referred to as ubiquitin-receptors. These receptors are responsible for translating ubiquitin modifications into cellular phenotypes. Ubiquitin can be attached to target proteins as a single moiety (ubiquitin or mono-ubiquitin, monoUb), or as polyubiquitin (polyUb) chains. For polyubiquitination, the ubiquitin domains are most commonly linked through its Lys 48 (K48) or Lys 63 (K63) residue. The K48-linked polyUb chains adopt a kinked topology, whereas K63-linked polyUb chains are more linear in conformation and resemble a ‘beads-on-a-string’ structure [13], [14]. Ubiquitin-receptors that recognize the K48-linked polyUb chains recruit the modified proteins to the proteasome for degradation. In contrast, ubiquitin-receptors that bind to monoUb or Lys 63 linkages enable non-degradative signaling processes by recruiting mono-ubiquitinated or Lys 63-polyubiquitylated proteins to downstream protein complexes. K63-linked ubiquitination, for example, is used as a key signal transducer for the activation of NF-κB and cell survival. Linear-linked polyUb chain has a structure highly similar to that of K63-linked polyUb chain [15]. The linear-linked polyUb chain, in which the C-terminal Gly of ubiquitin is conjugated to the α-amino group of the N-terminal Met of the successive ubiquitin, can be generated by a unique ubiquitin ligase complex known as the linear ubiquitin chain assembly complex (LUBAC) [16]. However, the physiological role of the linear-linked polyUb remains largely unknown except that it can also act as a regulator for the activation of NF-κB [17].

The UBA domain is a small (∼40 residues) protein-protein interaction module that mediates ubiquitin-binding and thus enables host proteins to participate in ubiquitin-dependent signaling processes [18]. Structural studies indicate that a conserved hydrophobic patch on the UBA domain makes direct contact with ubiquitin. The three-dimensional structures of a number of UBA domains have been determined using NMR and x-ray crystallography [19]–[28]. Despite remarkably low sequence homology, their three-dimensional structures are highly similar, consisting of a bundle of three α-helices. A majority of UBA domains bind ubiquitin or ubiquitin-like domains using a hydrophobic patch of residues in the α1–α2 loop (‘MGF’) and two aliphatic residues at the end of the helix α3 (‘LL/V’) [29], [30]. In spite of structural similarity, different types of UBA domain show distinct monoUb and linkage selective polyUb-binding ability [31]. Most studies reported so far describe UBA domain binding to monoUb as a heterodimer with a stoichiometry of 1 to 1. Self-association has been reported previously for a few cases of UBA and UBA-like domains [32]–[37]. However, only a few of them, such as Cbl-b-UBA [35], c-Cbl-UBA [34] and p62-UBA [36], have been structurally characterized.

Gyrd-Hansen *et al.* demonstrated with glutathione S-transferase (GST) pull-down assays that the UBA domain-containing IAPs do not associate with monoUb but they bind efficiently to K63-linked polyUb and in some cases to K48-linked polyUb [11]. In particular, XIAP binds exclusively to the structurally similar K63-linked and linear-linked polyUbs [11]. Furthermore, it was shown that the presence of a UBA domain and a dimerizable RING domain are essential for XIAP's ability to bind polyUb [11]. However, in a later report, Blankenship *et al.* demonstrated that the UBA domain of cIAP-1 binds monoUb and K48-linked and K63-linked polyUb chains with low-micromolar affinities by using multiple detection methods, including surface plasmon resonance, isothermal titration calorimetry and NMR spectroscopy [12]. Nevertheless, these novel observations establish IAPs as ubiquitin interacting proteins and highlight the need for a better understanding of the interactions between the UBA domains of IAPs with ubiquitin at molecular and structural levels.

In the present study, we have determined the solution structure of the UBA domain in XIAP by NMR spectroscopy, which is the first reported structure of a UBA domain in the IAP-family. In spite of a marked overall conformational similarity with reported UBA domain structures from other protein families, XIAP-UBA exhibits notable structural differences in the helical length and inter-helical packing as well as the presence of an N-terminal 3_10_ helix. Using a combination of chemical shift perturbation mapping and site-directed mutagenesis, we have elucidated the binding interfaces for ubiquitin as well as K48-linked and linear-linked diubiquitins (Ub_2_) on the XIAP UBA domain. In addition to the characterization of the hydrophobic ubiquitin-binding surfaces on XIAP-UBA, the present study provides evidence for the dimerization of the XIAP UBA domain. Mapping of the self-association surface of XIAP UBA domain reveals that it is formed by residues in N-terminal 3_10_ helix, helix α1 and helix α2, and is separate from the ubiquitin-binding surface. Our results provide a plausible model to explain how the self-association of XIAP UBA domain could lead to the linkage-specificity of polyubiquitin chain binding by XIAP.

## Results

### Solution structure of XIAP-UBA

**Figure 1:**
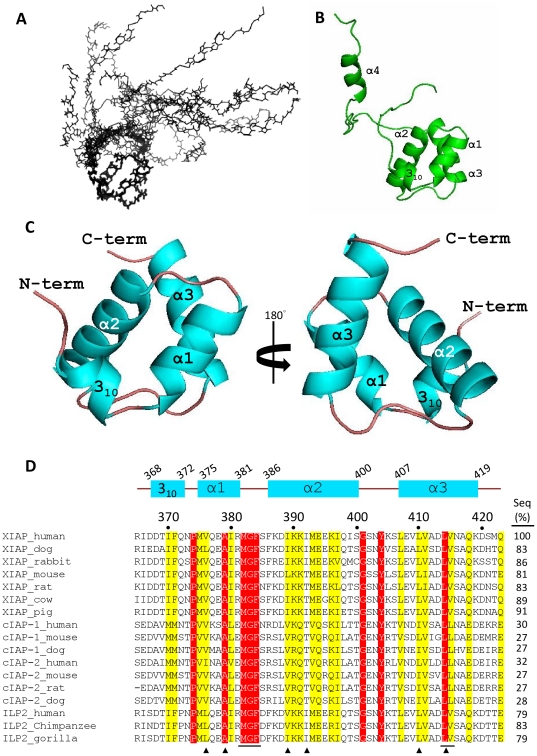
The solution structure of XIAP-UBA domain and sequence alignment of UBAs in IAP-family proteins. (A) The C_α_ trace of an ensemble of the 15 lowest energy NMR conformers for the XIAP-UBA-containing protein. Residues Asp368-Glu372 in helix 3_10_, Met375-Arg381 in helix α1, Phe386-Ser400 in helix α2 and Leu407-Lys419 in helix α3 were used for superimposition. (B) Ribbon presentation of the XIAP-UBA-containing protein of the lowest energy structure. (C) Ribbon presentation of the XIAP-UBA domain region. The secondary structure elements are labeled, with helix and loop colored cyan and brown, respectively. Panels A–C were generated by PyMOL. (D) Sequence alignments of UBA domains in mammalian IAP-family proteins. Invariant and partially conserved residues are highlighted in red and yellow, respectively. Residues that participate in the hydrophobic core formation in XIAP-UBA are marked with (▴). The putative ubiquitin-binding motif is underlined at the bottom of the alignment. The positions of the secondary structure elements are depicted at the top of the alignment. The protein similarities between XIAP-UBA and various IAP-UBAs are listed at the right of the alignments. All protein sequences were obtained from Swiss-Prot (www.expasy.ch), and they were analyzed in ClustalW2 (http://www.ebi.ac.uk/Tools/msa/clustalw2/).

The solution structure of the ^15^N/^13^C-labeled XIAP-UBA construct (residues 357–449) was determined from a total of 1357 NOE-derived distance restraints (Table 1). All the assignments of NOEs distance restraints, in particular for the interfacial residues (residues 369–373, 377, 378, 386, 383 and 381) (as described below), were carefully checked, and the structure determination at this stage assumed a monomeric structure. The final ensemble of 15 conformers was well defined (Figure 1A). The root-mean-square deviations (RMSDs) of backbone and heavy atoms over residues 368–419, which form the UBA domain core, were 0.31 Å and 0.71 Å, respectively. The C-terminal polypeptide segment (residues 420–449), which includes a helix (α4; residues 437–443), was not well converged in the ensemble because no long-range NOEs for this segment were observed (Figure 1B). This C-terminal segment includes part of the XIAP-RING domain (residues 434–497) [11] and a flexible region (residues 420–437), and played no detectable role in respect of the protein association studies reported here (data not shown). The mean coordinates of the core XIAP UBA domain encompassing residues 365–423, denoted XIAP-UBA in the following text, is selected for further structure description (Figure 1C).

**Table 1:** NMR structure determination statistics.

**NOE-based distance constraints**	
Short range (|i−j|≤1)	732
Medium range (1<|i−j|<5)	325
Long range (|i−j|≥5)	300
Total	1357
**Dihedral angle (ϕ/ψ) constraints from TALOS: ϕ+ψ**	53+53
**Hydrogen bonds**	42
**Violations**	
NOE distance violations ≥0.5Å	0
Torsion angles ≥5°	0
**Average Van der Waal energy (kcal/mol)**	−613.2
**Mean RMS deviations from the average coordinate (Å)** #	
Backbone atoms	0.31
All heavy atoms	0.79
**Ramachandran plot statistics for ϕ and ψ angles**	
Residues in most favored region (%)	88.7
Residues in additional allowed region (%)	11.3
Residues in generously allowed region (%)	0.0
Residues in disallowed region (%)	0.0

# computed values included residues 368–419 of XIAP.

The XIAP-UBA adopts a compact globular three-helix bundle structure, which highly resembles the classical UBA domain topology. The three helices (α1, α2 and α3) are packing against each other to form a well-defined hydrophobic core, which is composed of residues Val376, Ala379, Ile389, Ile392, Leu410 and Leu414. In addition, XIAP-UBA possesses an N-terminal 3_10_ helix (residues 368–372) oriented nearly perpendicular to helix α1 (interhelical angle ∼98°). This N-terminal short helix is so far reported only in Dsk2-UBA (helix α0) where its function remains uncharacterized.

A DALI search for the structural homologues of XIAP-UBA returned five hits with a Z-score >3.0, including Dsk2-UBA (Z-score = 3.8, Cα RMSD = 2.2 Å), hHR23A-UBA2 (Z-score = 3.6, Cα RMSD = 2.9 Å), Cbl-b-UBA (Z-score = 3.5, Cα RMSD = 2.4 Å)[35], Ede1-UBA (Z-score = 3.3, Cα RMSD = 2.2 Å) and UQ1-UBA (Z-score = 3.1, Cα RMSD = 2.8 Å) [38]. The DALI results indicate that the overall structure of XIAP-UBA is well-conserved even though the sequence similarities between XIAP-UBA and the returned hits are very low (6–11%) (Figure S1).

### Mapping of the mono-ubiquitin, K48-linked and linear-linked diubiquitin binding sites on XIAP-UBA

NMR chemical shift perturbation (CSP) experiments were used to map the interacting surface of XIAP-UBA with monoUb, K48-linked diubiquitin (K48-Ub_2_) and linear-linked diubiquitin (LL-Ub_2_). All the studies were performed using a protein construct comprising residues 365–423 of XIAP (XIAP-UBA). CSPs for ^15^N-labeled XIAP-UBA were monitored in a series of ^1^H-^15^N HSQC spectra where unlabeled monoUb, K48-Ub_2_ and LL-Ub_2_ were respectively titrated in small increments until saturation (i.e. final [monoUb]: [XIAP-UBA] = 5.5, [K48-Ub_2_]: [XIAP-UBA] = 2.5 and [LL-Ub_2_]: [XIAP-UBA] = 2.5).

**Figure 2:**
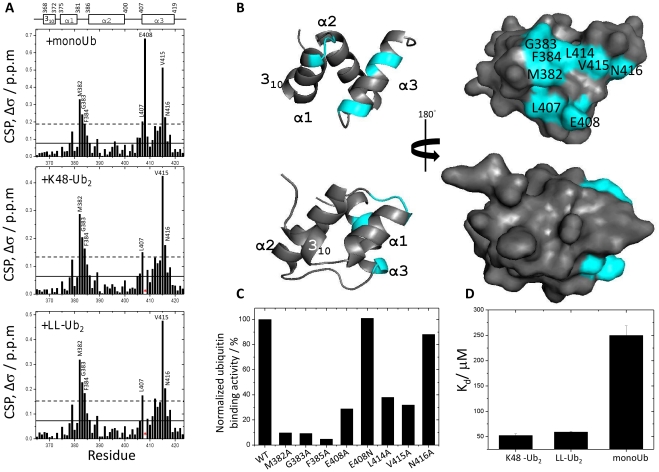
Mapping of mono-ubiquitin and diubiquitins binding interfaces on XIAP UBA domain. (A) The residue-wise chemical shift perturbation (CSP) in XIAP-UBA upon the addition of saturated amounts of mono-ubiquitin, (top panel), K48-linked diubiquitin (middle panel) and linear-linked diubiquitin (bottom panel). The average (solid line) and the average plus one standard deviation (S.D.) (dotted line) of CSP values are indicated. Residues that exhibited peak broadening are marked by red asterisks. The secondary structures along the sequence are marked at the top. (B) Surface map of significantly perturbed residues in XIAP-UBA upon mono-ubiquitin binding. The residues with a CSP value higher than one S.D. above the average were considered as significantly perturbed and labeled in this panel. The panel was generated using PyMOL (DeLano Scientific). (C) XIAP-UBA mutants have reduced the ubiquitin-binding affinities as detected by NMR spectroscopy. (D) The measured dissociation constants (K_d_) of interactions between XIAP-UBA and mono-ubiquitin, K48-linked diubiquitin and linear-linked diubiquitin respectively.

During the titration with unlabeled monoUb, the positions of backbone amide resonances of ^15^N-XIAP-UBA were observed to move progressively, consistent with a typical two-state fast exchange binding process. Significant CSPs were detected for residues between Met382 to Phe384 (α1–α2 loop), Leu407 to Glu408 (N-terminal region of helix α3) and Val415 to Asn416 (C terminal region of helix α3) (Figure 2A, upper panel). All of these residues map to a contiguous surface on XIAP-UBA (Figure 2B). It should be noted that residues Met382 to Phe384, and Leu414 to Val415 are invariantly conserved throughout the IAP-family (Figure 1D), forming a putative ubiquitn-binding motif [11]. Alanine substitution of Met382, Gly383, Phe384, Leu414 and Val415 caused a significant reduction of ubiquitin-binding to XIAP-UBA, and supports the direct involvement of these residues in ubiquitin-UBA interaction (Figure 2C). Although a significant CSP (Δσ = 0.22 ppm) was observed for residue Asn416, the N416A mutant only loses partial ubiquitn-binding activity. The result suggests that Asn416 plays a less critical role in the interaction with ubiquitin (Figure 2C). The observed large CSP on Asn416 upon ubiquitin binding likely results from conformational effect rather than direct interaction with ubiquitin. Interestingly, the resonance of a semi-conserved residue, Glu408 (position equivalent to Asn428 of cIAP-1 and Asn414 of cIAP-2 [12]), exhibited the largest CSP value (Δσ = 0.68 ppm). Mutation of Glu408 to an Asn had no adverse effect on ubiquitin binding activity. In contrast, alanine substitution of Glu408 substantially reduced the interaction with ubiquitin (Figure 2C). These results imply that the polar side-chain of Glu408, which protrudes from the ubiquitin-binding surface, is important for the interaction (Figure 2B).

The K48-linked and linear-linked diubiquitin-induced chemical shift perturbation profiles of ^15^N-XIAP-UBA were remarkably similar to that induced by mono-ubiquitin, with significant perturbations observed for Met382-Phe384, Leu407, Leu415 and Asn416 (Figure 2A, middle and lower panels). In addition, residue Glu408 also experiences strong signal attenuation and line broadening, indicative of an intermediate exchange regime on the NMR timescale and its involvement of intermolecular interface. The present CSP results suggest that XIAP-UBA interacts with K48-Ub_2_ and LL-Ub_2_ through the same molecular surface as in the XIAP-UBA-monoUb interaction. Similar observations have also been reported in other UBA/monoUb pairs beyond the IAP-family proteins [38], [39]. Quantitative analysis of the CSPs as a function of added monoUb, K48-Ub_2_ and LL-Ub_2_ yielded dissociation constants (K_d_) of 249±19 µM, 52±3 µM and 59±11 µM, respectively (Figure 2D).

### Mapping of XIAP-UBA binding sites on mono-ubiquitin

**Figure 3:**
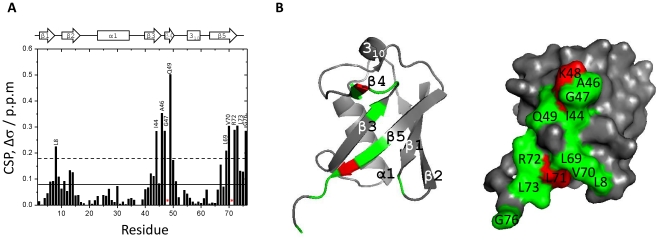
Chemical shift perturbation mapping of XIAP-UBA binding site on mono-ubiquitin. (A) The residue-wise chemical shift perturbation (CSP) in ubiquitin upon addition of saturating amounts of XIAP-UBA. The average (solid line) and the average plus one standard deviation (S.D.) (dotted line) of CSP values are marked. Residues that exhibit peak broadening are marked by red asterisks. The secondary structures along the sequence are indicated at the top. (B) Surface map of significantly perturbed residues in XIAP-UBA upon mono-ubiquitin binding. Residues with a CSP value higher than one S.D. above the average was considered as significantly perturbed and are colored green. Residues whose cross peaks showed broadening are colored. The mono-ubiquitin structure was retrieved from Protein Data Bank (PDB code: 1UBQ). The panel was generated using PyMOL (DeLano Scientific).

To identify the binding site of XIAP-UBA on monoUb, CSP mapping experiments were also performed using ^15^N-enriched monoUb and unlabeled XIAP-UBA proteins. Substantial perturbations were observed in three structural regions surrounding the residues near Leu8 (β1–β2 loop), Ile44 (strand β3) and Val70 (strand β5) in ubiquitin (Figure 3A and 3B). This result was consistent with previous reports that the Leu8-Ile44-Val70 hydrophobic patch forms a common binding site for UBA and other ubiquitin-binding domains (UBD). Intriguingly, significant perturbation (Δσ = 0.28 ppm) was also detected for Gly76. Residues Gly75 and Gly76 constitute the C-terminal diglycine motif, which has not been involved in UBA-monoUb interactions before. This suggests a potential role of Gly76 in monoUb-XIAP-UBA interaction.

### Mapping of self-association surface on XIAP-UBA

**Figure 4:**
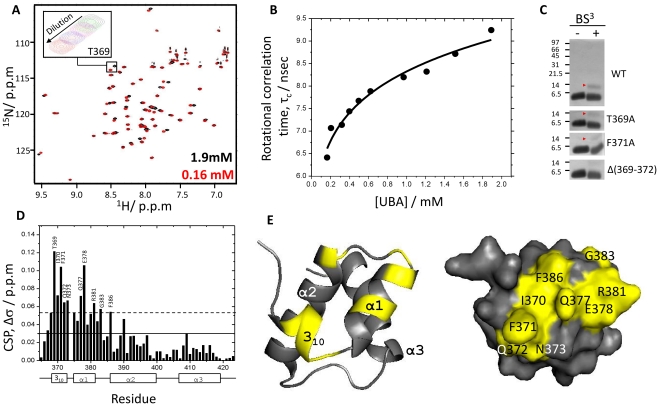
Concentration-dependent self-association of XIAP-UBA domain. (A) Overlay of ^1^H-^15^N HSQC spectra of 0.16 mM (red) and 1.9 mM (black) XIAP-UBA. Inset: An expanded region of the spectra indicates that ^1^H-^15^N resonance of Thr369 moves in a concentration dependant manner. (B) Rotational correlation time (τ_c_) values at different XIAP-UBA concentrations. (C) SDS-PAGE analysis of XIAP-UBA crosslinking products. XIAP-UBA and its mutants were incubated in the presence of the homo-bifunctional crosslinker BS^3^, followed by SDS-PAGE analysis. Molecular mass markers are indicated on the left. The dimeric species are indicated by the red arrows. (D) The residue-wise combined ^15^N and ^1^H chemical shift changes in XIAP-UBA upon dilution. The changes were calculated as CSPs, as described in Materials and Methods. The average CSP is represented by a solid line while the average CSP plus one standard deviation (S.D.) by a dotted line. The secondary structures along the sequence are indicated at the bottom. (E) Mapping of significantly perturbed residues on the XIAP-UBA upon concentration change. The figure was generated using PyMOL (DeLano Scientific).

A comparison of the 2D ^1^H-^15^N HSQC spectra recorded on ^15^N- labeled XIAP-UBA at different concentrations (0.16 mM to 1.9 mM) revealed a series of changes in cross peak position (Figure 4A). This observation suggests the presence of an oligomerization equilibrium in the fast exchange regime. The self- association of XIAP-UBA was further supported by the measurement of its rotational correlation time (τ_c_) at different concentrations. The rotational correlation time of XIAP-UBA was estimated from backbone ^15^N longitudinal and transverse relaxation times, using pseudo-2D ^15^N-edited T_1_ and T_2_ gradient experiments, respectively, at 298 K (see Materials and Methods) and was found to grow with increasing protein concentration (Figure 4B). The τ_c_ values for XIAP-UBA were 6.4 ns and 9.3 ns at 0.16 mM and 1.9 mM, respectively. Using in-house ^15^N-labeled monomeric protein calibration standards, the effective molecular masses were estimated to be 7.3 kDa and 12.9 kDa for τ_c_ values of 6.4 ns and 9.3 ns, respectively. Given that the predicted monomer molecular mass of XIAP-UBA is 7.2 kDa, the analysis of rotational correlation time is in accord with a shift in the monomer-dimer equilibrium towards the dimeric state with increasing protein concentration. At the highest XIAP-UBA concentration tested (1.9 mM), the estimated molecular weight of protein (12.9 kDa) is smaller than that of theoretical value of XIAP-UBA dimer (14.4 kDa). The observation is explained by the fact that the measured τ_c_ value represents the population-weighted average of the predominant dimeric species and the sparsely populated monomeric species. Fitting by non-linear curve fitting of the τ_c_ variation yielded a dimer dissociation constant of ∼900 µM. The existence of a monomer-dimer equilibrium in solution was further supported by protein cross-linking experiments and SDS-PAGE analysis (Figure 4C). A gel band corresponding to dimeric cross-linked XIAP-UBA (∼14 kDa) was detected upon the addition of bis(sulfosuccinimidyl) suberate cross-linker (BS^3^) (Figure 4C, upper panel, red arrow).

To dissect the location of the dimeric interface, the CSP mapping approach was applied to monitor the changes in chemical shifts at different XIAP-UBA concentrations. Significant perturbations were detected for Thr369-Asn373 (3_10_ helix and 3_10_-α1 loop), Gln377-Glu378 (helix α1), Arg381 (helix α1), Gly383 (α1–α2 loop) and Phe386 (helix α2) (Figure 4D). Mapping of the CSPs on the XIAP-UBA monomer structure yields a contiguous binding surface, which is comprised of both hydrophobic and polar residues (Figure 4E). This oligomerization site is distinct from the ubiquitin-binding site (Figure 2B). In order to test the contribution of the 3_10_ helix to the self-association, mutant XIAP-UBA proteins, namely T369A and F371A, were respectively subjected to BS^3^ cross-linking as described above. These two XIAP-UBA mutants were still able to be cross-linked into homodimers, although the cross-linking efficiency was apparently weaker than for the wild type (WT) protein. Furthermore, deletion of the 3_10_ helix in the XIAP-UBA mutant, Δ (369–372), abolished dimer formation (Figure 4C). These results indicate that the N-terminal 3_10_ helix is an important constituent of the surface responsible for self-association.

### Molecular docking of monoUb-binding and dimerization of XIAP-UBA

Models for the XIAP-UBA/ubiquitin complex and XIAP-UBA/XIAP-UBA homodimer were generated using the restraints-driven docking program HADDOCK v2.0 [40], [41]. CSPs derived from the NMR experiments were translated into ambiguous interaction restraints (AIRs), which were used to drive the docking process (see Material and Methods).

**Figure 5:**
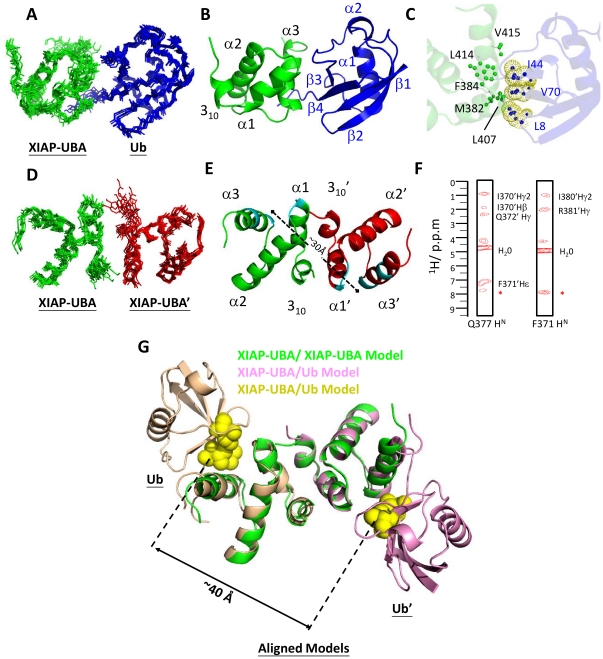
Models of the XIAP-UBA dimer, XIAP-UBA/Ub complex and [XIAP-UBA/Ub] _2_ tetrameric complex. (A) and (D) C_α_ traces of the ensemble of the 10 lowest energy HADDOCK structures of XIAP-UBA/Ub and the XIAP-UBA homodimer. (B) and (E) Ribbon representation of the ensemble-averaged HADDOCK structures of XIAP-UBA/Ub and XIAP-UBA dimer. The center-of-mass distance between the ubiquitin-binding sites (cyan) were shown in (E). (C) Contacts at the XIAP-UBA/monoUb interface showing the burial of the key hydrophobic side chains from the ubiquitin-binding site (green) of XIAP-UBA and Leu8-Ile44-Val70-centred hydrophobic patch (blue with yellow dotted spheres) of ubiquitin. Residues and secondary elements in one half of the XIAP-UBA homodimer are denoted with a prime to distinguish them from another half of the dimer. (F) Partially assigned ^1^H-^1^H strips for backbone amide of Gln377 and Phe371 of XIAP-UBA showing intermolecular NOEs from the ^13^C, ^15^N F1-filtered, ^15^N-F3-edited 3D NOESY-HSQC spectrum of the half-labeled XIAP-UBA dimer. The position of the diagonal peak is indicated by a red asterisk. (G) Ribbon representation of the aligned models. The center-of-mass distance between the Leu8-Ile44-Val70-centred hydrophobic patches (yellow sphere) is shown. Ubiquitin domains on the XIAP-UBA and XIAP-UBA′ are labeled as Ub and Ub′ respectively. All figures (except (F)) were generated using PyMOL (DeLano Scientific).

A model complex of XIAP-UBA and mono-ubiquitin (XIAP-UBA/Ub) was calculated using the chemical shift restraints derived from the NMR titration data (Table S2 and Figure 2A and 3A). The lowest-energy HADDOCK cluster contains 45 conformers with backbone RMSD of 1.2±0.9 Å (Table S3). An ensemble of ten structural models from this cluster showed a well defined complex with a consistent orientation of the ubiquitin molecule (labeled as Ub in Figure 5A & 5B). The structure of the XIAP-UBA/Ub model closely resembles to other known UBA/monoUb complex (RMSD for Cα atoms ∼1.8–2.1 Å) (Figure S2). The model shows that residues in the ubiquitin-binding binding motif of XIAP-UBA (Met382, Gly383, Phe384, Leu414 and Val415) and Leu407 make multiple hydrophobic contacts with residues Leu8, Ile44 and Val70 in the hydrophobic patch of monoUb (Figure 5C) in a manner fully consistent with our mutagenesis results.

We have also generated models of the XIAP-UBA homodimer (XIAP-UBA/XIAP-UBA′) with HADDOCK using the concentration-dependant CSP data of XIAP-UBA (Table S1 and Figure 4D). An ensemble of 10 structural models from the lowest-energy HADDOCK cluster is shown in Figure 5D and 5E. This cluster consists of 181 conformers, with average pairwise RMSD 1.2±0.8 Å for backbone atoms (Table S3). The homodimeric XIAP-UBA models bury 1328±54 Å^2^ of surface area, which is consistent with a recent survey that estimated the size of weakly-associated homodimeric interfaces at 1620±480 Å^2^ [42]. In the present model, each XIAP-UBA monomer uses its N-terminus and 3_10_ helix regions to bind a surface of the complementary monomer (XIAP-UBA′) comprising the region of helix α1′ in a reciprocal pairwise fashion. The interaction leads to the burial of hydrophobic residues, including Ile366, Ile380 and Phe386 from each chain.

The existence of close interaction between the 3_10_ helix of one monomer and the helix α1 from the complementary monomer is supported by the observation of intermolecular NOEs in a ^15^N-F3-edited 3D NOESY-HSQC spectrum (partially assigned ^1^H-^1^H strips shown in Figure 5F)[43]. It should be noted that only a small number of intermolecular NOEs at the dimer interface could be detected and assigned unambiguously. As shown in Figure 5F, intermolecular NOEs between the backbone amide of Gln377 in helix α1 and the side-chain methyl group of Ile370′ and aromatic ring of Phe371′ in helix 3_10_′ were observed. In addition, intermolecular NOE correlations between backbone amide of Phe371 and the side-chain of Arg381′ and methyl group of Ile370′ in helix α1′, were detected. These unambiguously assigned intermolecular NOEs provide direct evidences of the self-association of XIAP-UBA and support the close proximity of helix 3_10_ of one monomer and helix α1 of the complementary monomer at the dimer interface.

A noteworthy feature of the XIAP-UBA model is the geometric arrangement of the ubiquitin-binding sites (Met382 to Phe384, Leu407-Glu408, and Leu414-Val415) on the opposite sides (Figure 5E). The center-of-mass distance between these two sites in the lowest-energy HADDOCK cluster is 30±0.6 Å, sufficient to accommodate two molecules of ubiquitin each with radius of gyration of about 11 Å. The resulting model implies that the geometric arrangement of ubiquitin-binding and self-association surfaces would allow simultaneous binding of ubiquitin to both protomers in the XIAP-UBA dimer. We generated a hypothetical tetrameric [XIAP-UBA/Ub] _2_ complex model by performing structural superposition of two XIAP-UBA/Ub models (Figure 5B) on a single XIAP-UBA/XIAP-UBA′ homodimer (Figure 5E). The backbone pairwise RMSD values for the alignments of the XIAP-UBA domain of XIAP-UBA/Ub to the XIAP-UBA and XIAP-UBA′ of the XIAP-UBA/XIAP-UBA′ dimer were 0.532 Å and 0.432 Å, respectively. The tetramer model shown in Fig. 5G illustrates that there is a marked separation between the two mono-ubiquitin subunits. When a similar exercise was performed for the entire ensemble of HADDOCK dimer models, the average center-of-mass distance between the two Leu8-Ile44-Val70 hydrophobic patches of ubiquitin was 40±0.4 Å.

## Discussion

So far, no structure has been reported for the newly identified UBA domains of the IAP family proteins. The UBA domains share high sequence identities among human IAP family proteins (30–79%, Figure 1D), which implies that they possess conserved structure and function. We have determined the solution structure of the UBA domain of XIAP, which is the first reported structure of UBA domain in the IAP-family [11]. Despite the lack of sequence homology among UBA domains from different protein families (Figure S1A), the three dimensional structure of XIAP-UBA is similar to that of previously reported UBA domains, in which a globular three helix bundle topology is preserved (Figure S1B). In particular, the highly conserved residues (MGF…..LV/L) form a contiguous hydrophobic surface for ubiquitin interaction (Figure 2A and 2B). In spite of a marked overall conformational similarity, XIAP-UBA exhibits notable structural differences in the helix length and inter-helical packing. Structure comparison reveals that the lengths of helices α2 and α3 in XIAP-UBA are remarkably longer than those in the other reported UBA structures, including Dsk1-UBA [30], hHR23A-UBA2 [44], Cbl-b-UBA [35], Ede1-UBA [29] and UQ1-UBA [38] (Figure S1). The α1 helix, on the other hand, is relatively short due to the existence of an invariant proline Pro374 that serves as a helix breaker and demarcates the amino end of the helix α1 (Figure 1D). The inter-helical packing between helix α1 and helix α2, as well as between helix α1 and helix α3, forms more obtuse interhelical angles in XIAP-UBA (138° and 59°, respectively) compared with those in the other UBA structures. The interhelical angle between helix α2 and helix α3 is 102°, which is close to those observed in Swa2-UBA (102°) and hHR23A-UBA2 (103°) but considerably smaller than that observed in other UBA structures (ranging from 118° to 136°) (Table S1). In addition, XIAP-UBA contains an unusual N-terminal 3_10_ helix (residue 368–372) which is reported only in the UBA domain of Dsk2. Our CSP and mutation studies suggest that the 3_10_ helix is a crucial constituent of specific self-association surface of the XIAP-UBA domain.

Using a combination of chemical shift perturbation mapping and site-directed mutagenesis, we found that XIAP-UBA has two distinct surfaces available for ubiquitin-binding and self-association (Figure 2B and 4E). The ubiquitin-binding surface on XIAP-UBA is formed by the residues, (Met383-Gly383-Phe384 and Leu414-Val415), which have been collectively termed the ubiquitin-binding motif [11]. Our findings are consistent with the typical ubiquitin-binding patterns observed in the complex structures of ubiquitin with Dsk1-UBA, UQ1-UBA and Ede1-UBA, in which the ubiquitin-binding motif has extensive contacts with the Ile44-center hydrophobic patch on ubiquitin [24], [45]. Interestingly, our CSP data indicated that the Gly76 cross peak of ubiquitin is significantly perturbed upon XIAP-UBA binding. So far, it has been reported that the ubiquitin diglycine motif (Gly75–Gly76) is required for its interaction with zinc-finger ubiquitin binding domain of USP5, but is not involved in any UBA-ubiquitin recognition [24], [46], [47]. Self-association has been previously reported for a number of UBA and UBA-like domains [32]–[37]. However, only a few of UBA dimers, such as Cbl-b-UBA [35], c-Cbl-UBA [34] and p62-UBA [36], have been structurally characterized. Here, we have delineated the dimerization interface of XIAP-UBA which is formed by the residues mainly located in helix 3_10_ and helix α1. This interface is arranged differently from that of Cbl-b-UBA or c-Cbl-UBA, in which helix α2 and helix α3 were reported to provide the main contribution to the dimerization surface. In addition, the self-association and ubiquitin interfaces of XIAP-UBA are located at two essentially non-overlapping surfaces, which are also markedly different from that observed in p62-UBA [36].

Contrary to the previous study which reported that either XIAP or XIAP-UBA domain was unable to bind monoUb [11], we have detected a low affinity (K_d_ = 249±19 µM) for this interaction. The K_d_ value obtained here is similar to the reported K_d_ values for Mud1-UBA (K_d_ = 350±50 µM) [26], hHR23A-UBA2 (K_d_ = 400±100 µM) [44] and Swa2-UBA (K_d_ = 535±25 µM) [48]. The ubiquitin-affinity of XIAP-UBA is, however, significantly weaker than that of many reported UBAs, including Ede1-UBA (K_d_ = 83±9 µM)[29], Cbl-b-UBA (K_d_ = 53.1±3.1 µM) [49], UQ1-UBA (K_d_ = 24±6 µM)[38], Dsk2-UBA (K_d_ = 14.8±5.3 µM)[30] and cIAP-1 (K_d_ = 56±4 µM) [12]. Our result could explain the previous observation that substitution of XIAP-UBA with Cbl-b-UBA in full length XIAP increased its potency in NF-_Κ_B activation [11].

Previous studies indicated that UBA domains in the IAP family exhibit binding selectivity towards polyubiquitin chain linkages [11], [15]. In particular, full length XIAP and cIAP-2 are highly specific for K63-linked polyubiquitin, but not for K48-linked polyubiquitin [11]. Linear-linked diubiquitin was shown to be structurally similar to K63-linked diubiquitin, and XIAP was reported to interact with both linear-linked and K63-linked conjugates with a similar binding strength [11], [15]. An interesting aspect of our results is that the isolated XIAP-UBA domain alone was able to bind K48-linked diubiquitin, and it did not discriminate between K48-linked (K_d_ = 52±3 µM) and linear-linked (K_d_ = 59±11 µM) diubiquitin chains. Therefore, our results are consistent with Blankenship's report that the isolated UBA domain of cIAP-1 binds monoUb, K48-linked and K63-linked polyUb chains with similar affinities in the low-micromolar range [12]. It should be noted that our K48- and linear-linked diubiqutin-induced CSP profiles of XIAP-UBA were remarkably similar to that induced by monoUb. These observations suggested that monoUb, K48-Ub_2_ and LL-Ub_2_ bind to XIAP-UBA in a similar fashion. Our results imply that the structural features of a single XIAP-UBA domain are not sufficient to rationalize the selective recognition of the K63-linked (or linear-linked) polyubiquitin over K48-linked polyubiquitin chain reported for full-length XIAP.

**Figure 6:**
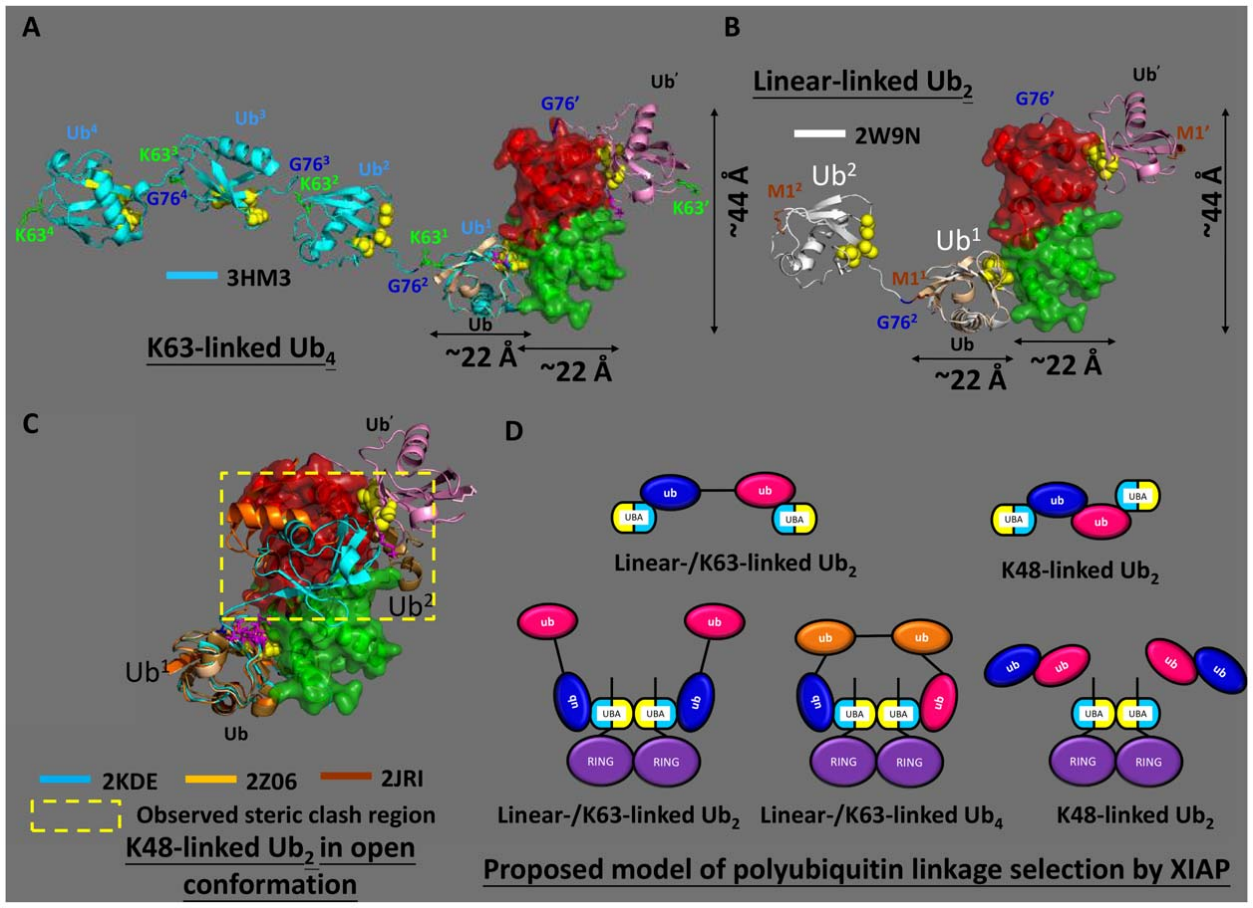
Superpositions of K48-, K63- and linear-linked polyubiquitins with the aligned model and the proposed mechanism of polyubiquitin linkage-selection by XIAP. Superpositions of the most proximal unit Ub^1^ of K63-linked tetraubiquitin (A, cyan ribbon (3HM3)), linear-linked diubiquitin (B, white ribbon (2W9N)) and K48-linked diubiquitin (C, cyan (2KDE), orange (2Z06), brown (2JRI) ribbons) with the ribbon structure of Ub (wheat) on the aligned models from Figure 5G. The Ub′ (pink ribbon) indicates the ubiquitin which associated with the XIAP-UBA′ protomer. The superscript prime denotes residues from Ub′. The superscript numbering refers to the position of the ubiquitin units in the polyubiquitin chain such that the Ub^1^ and M1^1^ denoted the most proximal unit and the Met1 of Ub^1^, respectively. The side-chains of residues M1 (brown), K63 (green) and G76 (blue) are labeled and shown in stick representation. The Leu8-Ile44-Val70-centred hydrophobic patch in ubiquitin is shown in yellow sphere. The XIAP-UBA and XIAP-UBA′ are shown in green and red semi-transparent surface. The region of steric clash between the Ub′ of the aligned models and the Ub^2^ of K48-linked diubiquitin is highlighted with a dotted rectangle in (C). (D) The speculative mechanism of polyubiquitin linkage-selection by XIAP. The XIAP-RING dimerization assists the oligomerization of XIAP-UBA, via the self-association (yellow) surface. The homodimeric XIAP-UBA arranges its ubiquitin-binding surfaces (cyan) into an orientation which can simultaneously recognize two separate (but not successive) ubiquitin units on the same flexible K63-/linear-linked polyubiquitin chain to achieve the high-affinity polyubiquitin chain binding (middle, bottom line). The binding of K48-linked polyubiquitin chain (in open conformation) with XIAP-UBA dimer is not spatially feasible (right, bottom line). Low-affinity binding events are observed between predominantly monomeric XIAP-UBA and the successive units of either K63-/linear-linked or K48-linked polyubiquitin. The observed subtle increase in binding affinity of diubiquitins to XIAP-UBA was probably due to increase of local concentration of ubiquitin in the diubiquitin chain (Top line). All figures were generated using PyMOL (DeLano Scientific).

The origin of polyubiquitin chain linkage selectivity by UBA-containing proteins remains to be elucidated. It was recently suggested that the orientations of the UBA domains, rather than the intrinsic structural properties of a single UBA domain, give rise to a diverse range of polyubiquitin linkage preference [39]. Here, we reported that XIAP-UBA has two independent surfaces for ubiquitin-binding and self-association. Our result suggests that the self-association of a single XIAP-UBA domain is weak (K_d_∼900 µM), but it is well known that XIAP exists as a dimer *in vivo* under the strong homodimerization influence of its RING and BIR1 domains [39], [50]. Therefore, it is reasonable to expect that the homodimerization of adjacent XIAP-RING domains at the C-terminus of XIAP could bring the two XIAP-UBA domains into close proximity and promote the formation of the UBA-UBA homodimer (Figure 6D). This prediction is supported by our preliminary evidence that the positions of ^1^H-^15^N HSQC cross-peaks corresponding to the residues at the dimerization interface (T369, F371, Q372, N373, Q377 and E378) were highly comparable between predominantly dimeric ^15^N-XIAP-UBA (1.9 mM) and dimeric ^15^N-XIAP-UBA-RING double-domain construct (0.18 mM) but very different from those of a predominately monomeric ^15^N-XIAP-UBA sample (0.16 mM) (Figure S3B). Furthermore, the 15.7 kDa ^15^N-XIAP-UBA-RING protein was detected as a dimer (apparent mass = 32.2 kDa) at a concentration as low as 0.18 mM using size-exclusion chromatography (Methods S1).

We speculate that the self-association of XIAP-UBA positions the ubiquitin-binding surfaces in a suitable orientation to simultaneously recognize two separate ubiquitin units on the same flexible K63- or linear-linked polyubiquitin chain but to discriminate against the binding of a K48-linked polyubiquitin chain (Figure 6D) [36], [51]. Our docking model of dimeric XIAP-UBA provides support for this proposal. The ubiquitin-binding sites are located on the opposite ends of the bowtie-shaped XIAP-UBA dimer. As illustrated in the superposed models (Figure 5G), the geometric arrangement of ubiquitin-binding sites allows simultaneous binding of two molecules of ubiquitin with a well-defined orientation to each other (labeled as Ub and Ub′ in Figure 6A–C). In the absence of binding partners, the K63-/linear-linked ubiquitin chain exhibits a flexible structure, in which the individual ubiquitin moieties can be regarded as independent rotationally unconstrained units. This structural plasticity allows a high degree of freedom to support simultaneous binding of two ubiquitin domains within the same polyubiquitin chain to a target containing two separated ubiquitin binding sites.

A superimposition of the most proximal ubiquitin (Ub^1^) of the K63-linked tetraubiquitin onto the ubiquitin (Ub in Figure 6A) of the aligned models reveals a marked spatial separation (∼22 Å) between the Gly76 of the succeeding Ub^2^ (Gly76^2^) of K63-linked tetraubiquitin and Gly76 of Ub′ (Gly76′) of the aligned models (Figure 6A). A similar separation has been observed in the superimposition of linear-linked diubiquitin structure onto the align models (Figure 6B). These observations imply that the simultaneous binding between the two successive ubiquitin units of polyubiquitin with the XIAP-UBA/XIAP-UBA′ appears to be spatially infeasible. Thus, for the simultaneous interactions of a XIAP-UBA dimer with two molecules of ubiquitin within the same chain, at least one and probably two ubiquitins are required as spacers to allow the most proximal and the distal ubiquitins to wrap around a XIAP-UBA dimer (molecular dimensions 44×22×22 Å) (Figure 6A and 6D). This model provides an explanation why the observed binding mode and affinity of linear-linked diubiquitin with dimeric XIAP-UBA is comparable to the low-affinity interaction between linear-linked diubiquitin and predominantly monomeric XIAP-UBA (K_d_∼micromolar, Figure 2D and 6D). Our model is further supported by the fact that dimeric full length XIAP requires a minimum of four ubiquitin domains (i.e. tetraubiquitin) for a detectable interaction [11]. It should be noted that, in the previous study by Gyrd-Hansen *et al.*, the interactions of XIAP with polyubiquitin chains of different linkages were investigated by conventional pull-down assay [11]. This technique is expected to detect relatively high affinity protein complexes with K_d_ in the sub-micromolar to nanomolar range [52].

In solution, K48-linked polyubiquitin chain exists in dynamic equilibrium between open and closed conformations. In the closed conformation, which is found predominantly in the absence of a binding partner, the K48-linked polyubiquitin chain has the Leu8-Ile44-Val70 hydrophobic patch of each ubiquitin unit buried in a compact conformation [53]–[55]. K48-linked diubiquitin is also known to shift to an open conformation upon binding to isolated hHR23A-UBA1 and hHR23A-UBA2 [39], [44]. Structural analysis of the hHR23A-UBA2/K48-Ub_2_ complex revealed that hHR23A-UBA2 contains an additional K48-Ub_2_ specific epitope, which is located on the opposite side to the ubiquitin-binding surface. Thus upon binding to hHR23A-UBA2, K48-linked diubiquitin wraps around the monomeric hHR23A-UBA2 and makes simultaneous hydrophobic contacts with the ubiquitin-binding surface and K48-Ub_2_ specific epitope [44]. Since no K48-Ub_2_ specific epitope is found on XIAP-UBA, its binding with K48-linked diubiquitin is presumably not favored and explains the observed low-affinity interaction between the predominantly monomeric XIAP-UBA and K48-linked Ub_2_ (Figure 2D and 6D). In addition, our aligned models provide further insight why K48-linked polyubiquitin does not interact with self-associated XIAP-UBA (Figure 6D). Superposition of the proximal ubiquitin (Ub^1^) of the K48-linked diubiquitin (in the open conformational state) onto the ubiquitin (Ub) of the aligned model reveals a severe steric clash between the distal ubiquitin (Ub^2^) and the complementary monomeric XIAP-UBA′ domain (Figure 6C).

## Materials and Methods

### Cloning and sample preparation

Two XIAP UBA protein constructs have been used in this study. The human XIAP-UBA -containing protein sequence, corresponding to Glu357-Leu449, was amplified via PCR and inserted into the pET-H expression vector as previously described [56]. This His-tagged construct was used primarily for NMR structure determination. For NMR titration, ^15^N-relaxation and protein cross-linking studies, GB1-His-tagged protein construct was prepared. The DNA sequence encoding the XIAP-UBA (Arg365-Gln423) was subcloned into pGB1-HIS bacterial expression vector. This construct encodes N-terminal GB1 and polyHis tags, which are separated from XIAP-UBA by a thrombin cleavage sequence (LVPRG). All XIAP-UBA mutants were prepared by site-directed mutagenesis of the wild-type GB1-His tagged expression construct using a QuickChange Site-Directed Mutagenesis Kit (Stratagene). All the DNA constructs were transformed in *E. coli* host BL21 (DE3) (Novagen) for recombinant protein expression. Unlabeled protein was prepared from cells grown in Luria-Bertani (LB) media. Uniformly labeled ^15^N or ^15^N/^13^C-labeled proteins were prepared from cells grown in M9 minimal media incorporating [U-^13^C]-glucose (Cambridge Isotopes Laboratories, CIL) and/or [U-^15^N]-ammonium chloride (CIL) as the sole carbon or nitrogen sources. The expression and purification of XIAP-UBA was essentially the same as described for that of XIAP-UBA-containing protein [56]. Unlabeled and uniformly ^15^N and ^15^N/^13^C-labeled samples of human ubiquitin were produced as described previously [49]. Unlabeled K48-linked and linear-linked diubiquitins were synthesized and prepared as described by Reyes-Turcu *et al* [57]. All protein samples were prepared in BisTris buffer (20 mM BisTris-HCl, pH 6.7, 150 mM NaCl, 5 mM d_10_-DTT, 1 mM PMSF, 90% H_2_O/10% D_2_O) for all NMR studies.

### NMR spectroscopy and structure calculation

NMR studies were performed at 298 K using a Bruker Advance 600 MHz or 700 MHz NMR spectrometer equipped with a TCI cyroprobe. NMR data were processed with TOPSPIN software (Bruker) and analyzed by SPARKY [58]. The backbone and side-chain resonance assignments of a 0.5 mM XIAP-UBA-containing protein (residues 357–449) were obtained using standard ^1^H/^13^C/^15^N heteronuclear NMR experiments as previously described [56]. The assignments were deposited in the Biological Magnetic Resonance Data Bank under accession number 16478 [56].

The solution structure of XIAP-UBA-containing protein (residues 357–449) was iteratively calculated using the CYANA protocols based on the NOE-derived inter-proton distance, backbone dihedral angle and hydrogen bonding constraints [59]. The inter-proton distance restraints were derived from 3D ^15^N-edited NOESY (mixing time = 120 ms) and 3D ^13^C-edited NOESY (mixing time = 120 ms) spectra, which were recorded separately on 0.45 mM ^15^N-labeled and 0.5 mM ^15^N/^13^C-enriched XIAP-UBA protein samples respectively. Cross-peaks in NOESY-type spectra were interactively assigned and integrated in XEASY. Backbone dihedral angle restraints were derived from chemical shift using TALOS [60]. Hydrogen bonding restraints in helical segment were determined on the basis of previously reported chemical shift index (CSI) values, dihedral angle values and characteristic NOE patterns. Out of 200 calculated conformers, the final coordinates for an ensemble of 15 lowest energy models were deposited into Protein Data Bank (PDB ID 2 kna), and they were used for subsequent structural analysis. Structure properties were analyzed using PROCHECK [61]. Interhelical angles were calculated in MOLMOL [62]. Structural similarity searches were performed with DALI using default settings [63]. All molecular graphic images were generated by using PyMOL(DeLano Scientific) [64].

NMR signal assignments of ubiquitin were taken from literature data [65].

The ^15^N relaxation experiments were performed using standard Bruker pseudo-2D ^15^N-edited T_1_ and T_2_ gradient pulse programs, hsqct1etf3gpsi3d and hsqct2etf3gpsi3d, respectively. Peaks in the amide proton region between 7.9–9.5 ppm were chosen for integration and analysis. Longitudinal and transverse relaxation times, T_1_ and T_2_, were obtained using the relaxation module of Topspin 2.1 (Bruker) as described in the user manual.

For the detection of intermolecular NOEs at the dimeric interface of XIAP-UBA, a mixed sample containing 1 mM ^13^C/^15^N-XIAP-UBA and 1 mM unlabeled XIAP-UBA was prepared in BisTris buffer. Intermolecular NOEs were recorded with a ^13^C, ^15^N F1-filtered, ^15^N-F3-edited 3D NOESY-HSQC experiment (mixing time = 200 ms) and ^13^C, ^15^N F1-filtered, ^13^C-F1-edited 3D NOESY-HSQC experiment (mixing time = 150 ms) as described previously [43].

### NMR titration studies of binding between UBA and mono-ubiquitin and diubiquitins

The mono-ubiquitin/diubiquitin binding surfaces on XIAP-UBA were mapped using the CSP approach. A series of 2D ^1^H-^15^N HSQC spectra of a ^15^N-labeled XIAP-UBA (0.5 mM) were recorded as a function of the increasing amount of unlabeled mono-ubiquitin, K48-linked diubiquitin and linear-linked diubiquitin, respectively. In general, 5 mM and 2.5 mM stock solutions of unlabeled mono-ubiquitin and diubiquitin were prepared, respectively. The NMR titration experiments were performed until the molar ratios of [monoUb]/[XIAP-UBA], [K48-Ub_2_]/[XIAP-UBA] and [LL-Ub_2_]/[XIAP-UBA] reached the values of 5.5, 2.5 and 2.5, respectively. In the final titration point, the final concentration of ^15^N-labled XIAP-UBA was around 0.3 mM, in which predominant monomeric XIAP-UBA was available for ubiquitin interaction. Binding was monitored through the changes in the cross-peaks positions of the ^1^H-^15^N HSQC spectra. These changes of cross-peak chemical shifts were quantified using combined amide CSP calculated as Δσ = [(Δσ_H_)^2^+(Δσ_N_/5)^2^]^1/2^, where Δσ_H_ and Δσ_N_ are the observed chemical shift changes for ^1^H and ^15^N dimensions, respectively. The binding affinities (dissociation constant, K_d_) between XIAP-UBA and ubiquitin/diubiquitin were quantified by fitting the CSPs as a function on the protein and unlabeled ligand concentrations to the appropriate stoichiometry/binding models as described [66] using ORIGIN 7.0 software (Origin Lab). To map the XIAP-UBA binding surface on mono-ubiquitin, a similar titration experiment was performed by adding unlabeled XIAP-UBA (2 mM stock solution) to ^15^N-labled mono-ubiquitin until the molar ratio of [XIAP -UBA]/[monoUb] was equal to 5.

The CSP approach was used to investigate ubiquitin-binding activity in wild type (WT) XIAP-UBA and XIAP-UBA mutants. ^15^N-labled monoUb was titrated with unlabeled XIAP-UBA, including WT or mutants, up to a [XIAP-UBA]: [monoUb] molar ratio of 5 as described above. At the final titration point, the average CSP value (AvgΔσ) of 12 well-resolved resonances in the ^1^H-^15^N HSQC spectrum of ubiquitin was calculated. The normalized percentage ubiquitin activity of each XIAP-UBA mutants was calculated as 100×[Avg(Δσ_mutant_)/Avg(Δσ_wild-type_)], where Avg(Δσ_mutant_) and Avg(Δσ_wild-type_) are the average CSP values obtained using XIAP-UBA and the corresponding XIAP-UBA mutant, respectively.

### Rotational correlation time measurement

The rotational correlation time (τ_c_) was determined to study the concentration-dependent oligomerization of XIAP-UBA. Dilution experiments were carried out over a range of XIAP-UBA concentration (0.16–1.9 mM). The 2D ^1^H-^15^N HSQC spectrum and the relaxation parameters (T_1_ and T_2_) were collected as described above. The (τ_c_) was calculated as τ_c_ = ((6T_1_/T_2_)-7)^1/2^/4πω_N_, where ω_N_ is the ^15^N resonance frequency (in Hz) [67]. A calibration plot (τ_c_ versus molecular weight) was generated using a series of in-house ^15^N-labeled standard monomeric proteins of known molecular weight (3–20 kDa).

The determination of dimer dissociation constant for XIAP-UBA was the same as described previously [35], except that non-linear regression analysis was performed for a plot of rotational correlation time as a function of protein concentration.

### Chemical cross-linking

Proteins were extensively dialyzed into 10 mM HEPES, pH 7.5 prior to cross-linking experiments using bifunctional crosslinker Bis (sulfosuccinimidyl) suberate (BS^3^) (Thermo scientific pierce). The XIAP-UBA wild type and mutant proteins (1 mg/ml) were treated with BS^3^ (500 µM) for 60 min at room temperature. The reactions were terminated by the addition of Tris salt (100 mM). Crosslinked products were separated by SDS-PAGE, followed by Coomassie blue staining.

### HADDOCK docking and analysis

The models of the XIAP-UBA/Ub complex and XIAP-UBA/XIAP-UBA′ homodimeric complex were calculated by using the HADDOCK web server (http://haddock.chem.uu.nl/services/HADDOCK) [40], [41]. The HADDOCK calculations of XIAP-UBA/Ub complex were started with the coordinates of human ubiquitin (PDB code: 1UBQ) and the averaged structure of the XIAP UBA domain (residues 365–423, PDB code: 2KNA). The starting structures for the docking calculation of XIAP-UBA/XIAP-UBA′ complex were two identical coordinates of the XIAP UBA domain (residues 365–423, PDB code: 2KNA).

The two docking procedures were driven by using ambiguous interaction restraints (AIRs), which were defined according to the CSP data obtained from NMR titration experiments of XIAP-UBA in complex with monoUb and from the dilution experiment of XIAP-UBA, respectively. Residues undergoing significant chemical shift perturbations (Δσ>1 S.D. above the mean value) were defined as active residues. Residues having a CSP value higher than the mean value but within 1 S.D. of the mean value were selected as a passive residue. The active and passive residues defined for the present calculations are summarized in Table S2. All other parameters were kept at the default settings.

The HADDOCK docking protocols principally consist of three stages, including a rigid-body energy minimization, a simulated annealing in torsion angle space allowing semi-flexibility, and an explicit water refinement. The web server returned 200 models, which were clustered according to the pair-wise RMSD matrix using a 5.0 Å cut-off. Ten different clusters have been obtained in the XIAP-UBA/Ub complex, while three different clusters have been identified in the homodimeric XIAP-UBA/XIAP-UBA′ models. These clusters were ranked according to their HADDOCK scores, which were defined as a weighted sum of van der Waals, electrostatic, solvation and restraint violation energy terms. The structural statistics of the top three clusters are shown in Table S3. In both docking runs, cluster 1 has the best average HADDOCK score. The 10 lowest energy structures from this cluster were selected as representative models of each docking complex. The aligned models were constructed as described in the text by using the structural alignment function of graphic program PyMOL (DeLano Scientific). The center-of-mass distance and molecular dimension (in terms of radius of gyration) of the representing model were calculated with PyMOL (DeLano Scientific). All structure superimpositions were performed using PyMOL (DeLano Scientific).

## Supporting Information

Figure S1 **Sequence alignment and superimposition of various UBA domains from different proteins.** (A) Sequence alignment of various UBA fold domains. The relative locations of secondary structure elements are boxed. The species of the primary sequences were as followed: Sc, *S. cerevisiae* (budding yeast); Sp, *S. pombe* (fission yeast); Hs, *H. sapiens* (human). The multiple sequence alignments were generated using ClustalW2. Summary of the output from the DALI server is shown on the right of the alignments. RMSD: Root-Mean-Square-Deviation for the structural alignment between the structures of corresponding UBA-fold protein and XIAP-UBA. Seq: Sequence similarity. (B) Superimposition Cα traces of XIAP-UBA with various UBA domains. Only the structures with a DALI Z-score >3.0 are selected for displayed. The color representation of each structure is indicated at the bottom. The PDB codes for structural alignment are as followed: Dsk2-UBA, 1WR1 (only structure available is in complex with ubiquitin); hHR23A-UBA2, 1DV0; UQ1-UBA, 2YJ5; Ede1-UBA, 2G3Q; Cbl-b-UBA, 2JNH. Structural alignment was performed using DALI server, and the image was created using PyMOL (DeLano Scientific).
(TIFF)

Figure S2 **Structural comparison among various UBA/monoUb complexes.** (A) The XIAP-UBA/Ub complex model obtained in docking study is aligned with the published solution structures of the ubiquitin complexes with the UBA domain of (B) UQ1 (PDB code: 2JY6), (C) Dsk2 (PDB code: 1WR1), and (D) Ede1 (PDB ID.: 2G3Q). The ubiquitin molecules are placed on the right side, the α-helixes and β-strands were colored in red/yellow and cyan, respectively; The UBA domains are placed on the left side, the 3_10_/α0, α1, α2 and α3 helixes are colored in red, green, blue and magenta, respectively. The image was created by MOLMOL (version 2K.1 by Reto Koradi).
(TIFF)

Figure S3 **Evidence of XIAP-RING-assisted homodimerization of XIAP-UBA as revealed by ^1^H-^15^N HSQC spectrum.** (A) Schematic representation of XIAP showing the protein domains used. (B) Overlay plots of ^1^H-^15^N HSQC spectra for the diluted sample of XIAP-UBA-RING (blue), the concentrated sample of XIAP-UBA (green) and the diluted sample of XIAP-UBA (red). For clarity, only the regions showing the resonances of dimerization interfacial residues are shown. Sample preparation and analytical size exclusion chromatography of XIAP-UBA-RING were described in Methods S1.
(TIFF)

Table S1 **Interhelical angles of various UBA domains.** ^#^The abbreviations of protein followed the description in the figure 2. The PDB code of each structure was followed: XIAP-UBA, 2KNA; UQ1-UBA, 2YJ5; hHR23A-UBA1, 1IFY; hHR23A-UBA2, 1DV0; Dsk2-UBA, 1WR1 (only structure available is in complex with ubiquitin); Ede1-UBA, 2G3Q; Mud1-UBA, 1Z96; Swa2-UBA, 1PGY; Cbl-b-UBA, 2JNH. ^$^The inter-helical angles were measured by the software MOLMOL (version 2K.1 by Reto Koradi) with the standard procedures in the manual. Firstly, the backbone atoms of a helix were selected, and a primitive of cylinder was added for the helix in spacing method by macro “AddCylinder spacing”. After cylinder was added for every helix, the cylinders (instead of the helices) were selected and the angles between the helix axes were calculated by the macro “CalcHelix”.
(DOC)

Table S2 **HADDOCK active and passive residue for construction of XIAP/Ub complex and XIAP-UBA/XIAP-UBA′complex.**
(DOC)

Table S3 **Summary of results from the top three clusters of docking result for the model complexes of XIAP-UBA/Ub and XIAP-UBA/XIAP-UBA′.** ^#^ Root-Mean-Square-Deviation from the overall lowest energy structure.
(DOC)

Methods S1 **Sample preparation of XIAP-UBA-RING and analytical size exclusion chromatography.**
(DOCX)
