# Supplementary figures and images for: Structural Analysis of the UBA Domain of X-linked Inhibitor of Apoptosis Protein Reveals Different Surfaces for Ubiquitin-Binding and Self-Association
Source: PLoS One. 2011 Dec 15;6(12):e28511. doi: 10.1371/journal.pone.0028511 (PMC3240630; doi:10.1371/journal.pone.0028511)

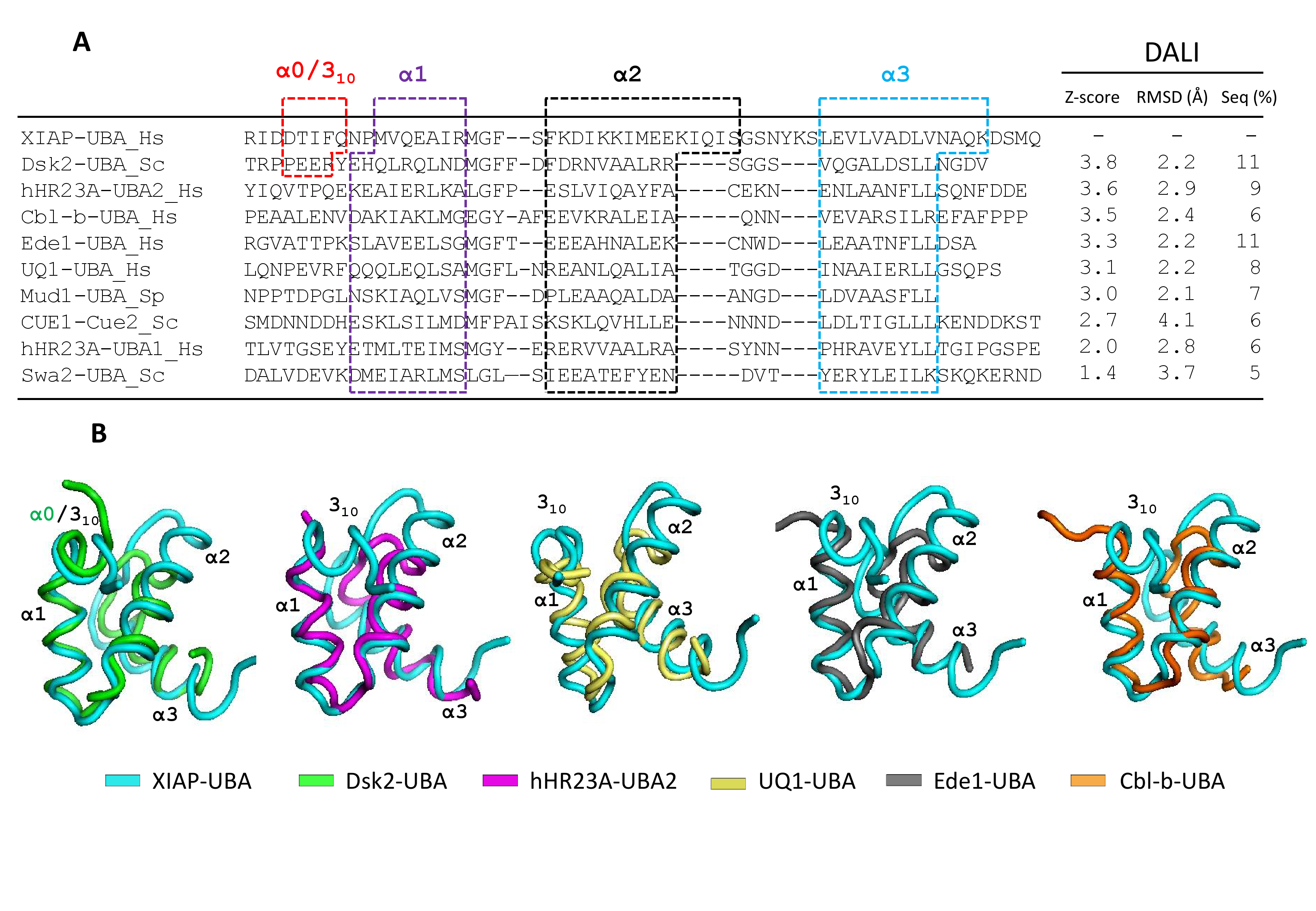

Supplement: Figure S1 — Sequence alignment and superimposition of various UBA domains from different proteins. (A) Sequence alignment of various UBA fold domains. The relative locations of secondary structure elements are boxed. The species of the primary sequences were as followed: Sc, S. cerevisiae (budding yeast); Sp, S. pombe (fission yeast); Hs, H. sapiens (human). The multiple sequence alignments were generated using ClustalW2. Summary of the output from the DALI server is shown on the right of the alignments. RMSD: Root-Mean-Square-Deviation for the structural alignment between the structures of corresponding UBA-fold protein and XIAP-UBA. Seq: Sequence similarity. (B) Superimposition Cα traces of XIAP-UBA with various UBA domains. Only the structures with a DALI Z-score >3.0 are selected for displayed. The color representation of each structure is indicated at the bottom. The PDB codes for structural alignment are as followed: Dsk2-UBA, 1WR1 (only structure available is in complex with ubiquitin); hHR23A-UBA2, 1DV0; UQ1-UBA, 2YJ5; Ede1-UBA, 2G3Q; Cbl-b-UBA, 2JNH. Structural alignment was performed using DALI server, and the image was created using PyMOL (DeLano Scientific). (TIFF) [file pone.0028511.s001.tiff]

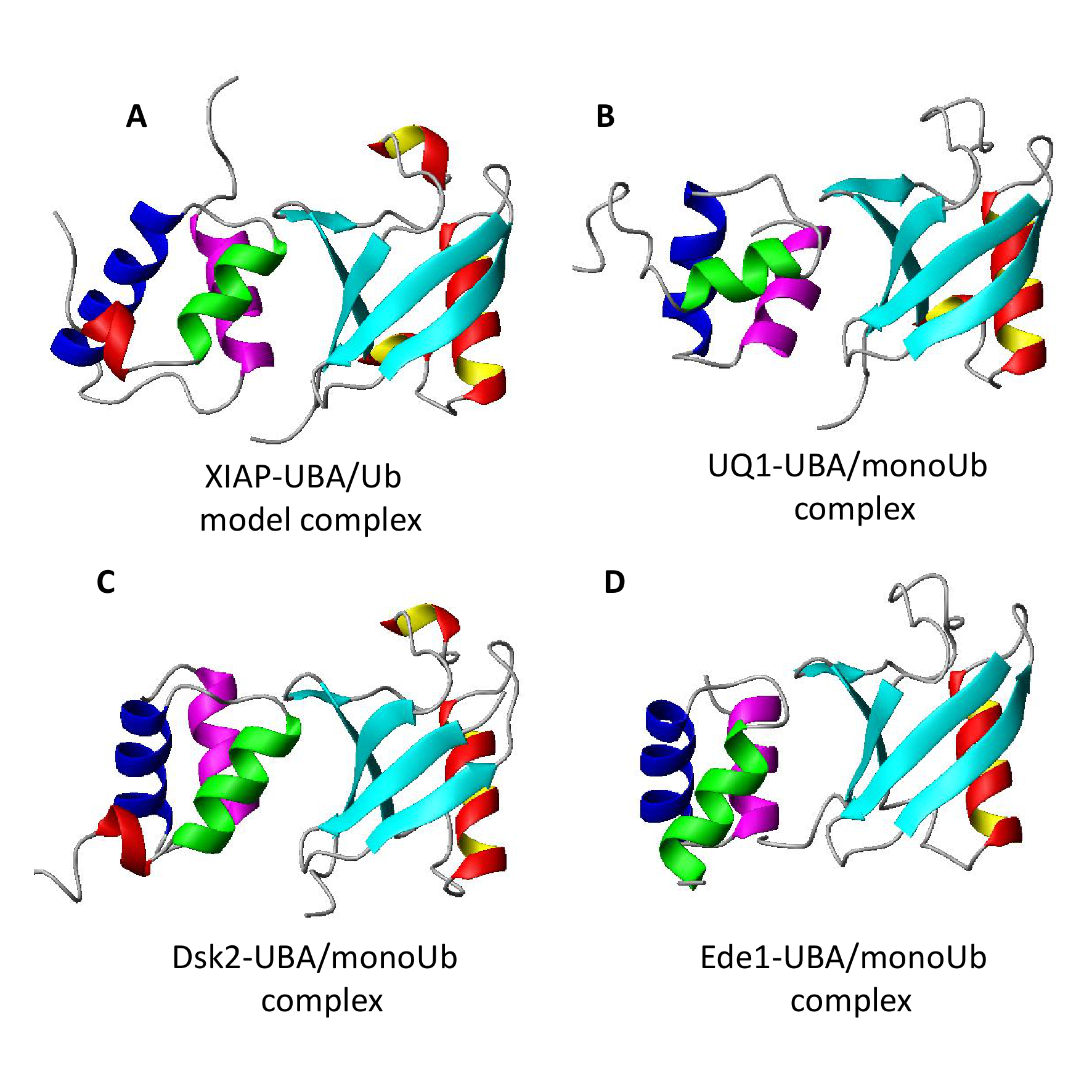

Supplement: Figure S2 — Structural comparison among various UBA/monoUb complexes. (A) The XIAP-UBA/Ub complex model obtained in docking study is aligned with the published solution structures of the ubiquitin complexes with the UBA domain of (B) UQ1 (PDB code: 2JY6), (C) Dsk2 (PDB code: 1WR1), and (D) Ede1 (PDB ID.: 2G3Q). The ubiquitin molecules are placed on the right side, the α-helixes and β-strands were colored in red/yellow and cyan, respectively; The UBA domains are placed on the left side, the 310/α0, α1, α2 and α3 helixes are colored in red, green, blue and magenta, respectively. The image was created by MOLMOL (version 2K.1 by Reto Koradi). (TIFF) [file pone.0028511.s002.tiff]

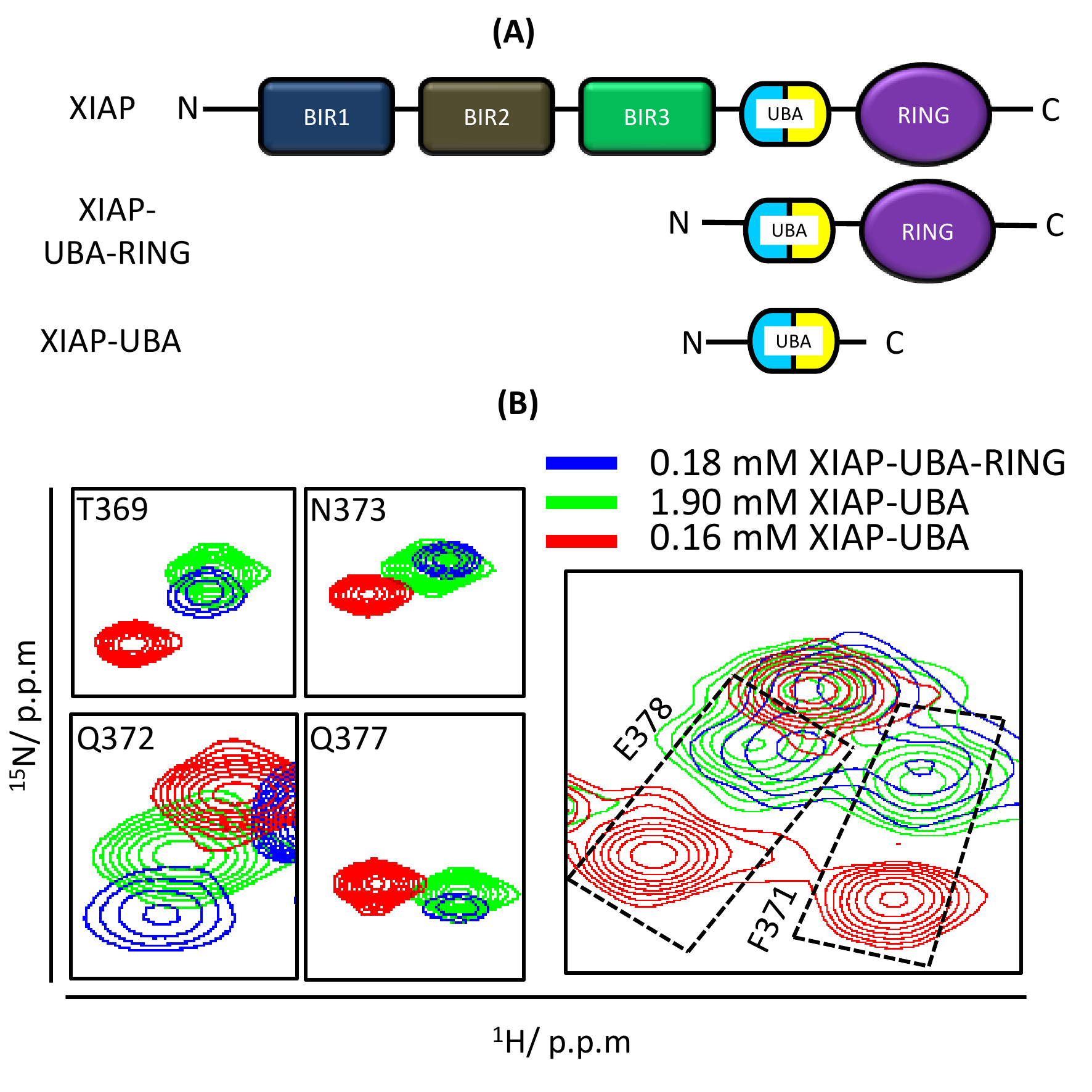

Supplement: Figure S3 — Evidence of XIAP-RING-assisted homodimerization of XIAP-UBA as revealed by 1H-15N HSQC spectrum. (A) Schematic representation of XIAP showing the protein domains used. (B) Overlay plots of 1H-15N HSQC spectra for the diluted sample of XIAP-UBA-RING (blue), the concentrated sample of XIAP-UBA (green) and the diluted sample of XIAP-UBA (red). For clarity, only the regions showing the resonances of dimerization interfacial residues are shown. Sample preparation and analytical size exclusion chromatography of XIAP-UBA-RING were described in Methods S1. (TIFF) [file pone.0028511.s003.tiff]
