# Supplementary material for: Structural Analysis of the UBA Domain of X-linked Inhibitor of Apoptosis Protein Reveals Different Surfaces for Ubiquitin-Binding and Self-Association
Source: PLoS One. 2011 Dec 15;6(12):e28511. doi: 10.1371/journal.pone.0028511 (PMC3240630; doi:10.1371/journal.pone.0028511)
Supplement: Table S1 — Interhelical angles of various UBA domains. #The abbreviations of protein followed the description in the figure 2. The PDB code of each structure was followed: XIAP-UBA, 2KNA; UQ1-UBA, 2YJ5; hHR23A-UBA1, 1IFY; hHR23A-UBA2, 1DV0; Dsk2-UBA, 1WR1 (only structure available is in complex with ubiquitin); Ede1-UBA, 2G3Q; Mud1-UBA, 1Z96; Swa2-UBA, 1PGY; Cbl-b-UBA, 2JNH. $The inter-helical angles were measured by the software MOLMOL (version 2K.1 by Reto Koradi) with the standard procedures in the manual. Firstly, the backbone atoms of a helix were selected, and a primitive of cylinder was added for the helix in spacing method by macro “AddCylinder spacing”. After cylinder was added for every helix, the cylinders (instead of the helices) were selected and the angles between the helix axes were calculated by the macro “CalcHelix”. (DOC) [file pone.0028511.s004.doc]

**Table S1. Interhelical angles of various UBA domains**

| Protein^#^ | Inter-helical angle (°)^$^ | | | | | |
| --- | --- | --- | --- | --- | --- | --- |
|  | 3_10_/α0-α1 | α0-α2 | α0-α3 | α1-α2 | α1-α3 | α2-α3 |
| XIAP-UBA | 98.0 | 88.6 | 154.3 | 137.7 | 58.6 | 102.0 |
| UQ1-UBA | - | - | - | 112.9 | 55.5 | 122.8 |
| hHR23A- UBA1 | - | - | - | 121.0 | 43.2 | 102.8 |
| hHR23A- UBA2 | - | - | - | 127.0 | 28.4 | 123.0 |
| Dsk2-UBA | 92.4 | 57.3 | 132.2 | 123.4 | 44.3 | 123.2 |
| Ede1- UBA | - | - | - | 128.0 | 34.3 | 117.7 |
| Mud1-UBA | - | - | - | 126.0 | 33.2 | 122.1 |
| Swa2-UBA | - | - | - | 102.9 | 23.1 | 101.7 |
| Cbl-b-UBA | - | - | - | 119.5 | 32.3 | 135.5 |

^#^The abbreviations of protein followed the description in the figure 2. The PDB code of each structure was followed: XIAP-UBA, 2KNA; UQ1-UBA, 2YJ5; hHR23A-UBA1, 1IFY; hHR23A-UBA2, 1DV0; Dsk2-UBA, 1WR1 (only structure available is in complex with ubiquitin); Ede1-UBA, 2G3Q; Mud1-UBA, 1Z96; Swa2-UBA, 1PGY; Cbl-b-UBA, 2JNH.

^$^The inter-helical angles were measured by the software MOLMOL (version 2K.1 by Reto Koradi) with the standard procedures in the manual. Firstly, the backbone atoms of a helix were selected, and a primitive of cylinder was added for the helix in spacing method by macro “AddCylinder spacing”. After cylinder was added for every helix, the cylinders (instead of the helices) were selected and the angles between the helix axes were calculated by the macro “CalcHelix”.
