# Supplementary material for: Structural Analysis of the UBA Domain of X-linked Inhibitor of Apoptosis Protein Reveals Different Surfaces for Ubiquitin-Binding and Self-Association
Source: PLoS One. 2011 Dec 15;6(12):e28511. doi: 10.1371/journal.pone.0028511 (PMC3240630; doi:10.1371/journal.pone.0028511)
Supplement: Table S2 — HADDOCK active and passive residue for construction of XIAP/Ub complex and XIAP-UBA/XIAP-UBA′complex. (DOC) [file pone.0028511.s005.doc]

**Table S2. HADDOCK active and passive residue for construction of XIAP/Ub complex and XIAP-UBA/XIAP-UBA’complex**

| **Model** | **XIAP-UBA/Ub** | | **XIAP-UBA/XIAP-UBA’** | |
| --- | --- | --- | --- | --- |
| **Structure**  **(PDB code)** | **XIAP-UBA**  **(2KNA)** | **Ubiquitin**  **(1UBQ)** | **XIAP-UBA**  **(2KNA)** | **XIAP-UBA**  **(2KNA)** |
| **Active residues** | M382, G383, F384, L407, E408, L414, V415, N416 | L8, I44, A46, G47, Q49, V70, R72, L73, G76 | T369, I370, F371, Q377, E378 | T369, I370, F371, Q377, E378 |
| **Passive residues** | S385, I389, K405, S406, V409, V411, A412, D413, Q418 | K6, T7, T9, K11, I13, T14, R42, L43, L50, E51, L67, L69, R74, G75 | Q372, N373, R381, G383, F386 | Q372, N373, R381, G383, F386 |
