# Supplementary material for: Structural Analysis of the UBA Domain of X-linked Inhibitor of Apoptosis Protein Reveals Different Surfaces for Ubiquitin-Binding and Self-Association
Source: PLoS One. 2011 Dec 15;6(12):e28511. doi: 10.1371/journal.pone.0028511 (PMC3240630; doi:10.1371/journal.pone.0028511)
Supplement: Table S3 — Summary of results from the top three clusters of docking result for the model complexes of XIAP-UBA/Ub and XIAP-UBA/XIAP-UBA′. # Root-Mean-Square-Deviation from the overall lowest energy structure. (DOC) [file pone.0028511.s006.doc]

**Table S3. Summary of results from the top three clusters of docking result for the model complexes of XIAP-UBA/Ub and XIAP-UBA/XIAP-UBA'**

| **Docking Model** | **XIAP-UBA/Ub** | | | **XIAP-UBA/XIAP-UBA’** | | |
| --- | --- | --- | --- | --- | --- | --- |
| **Cluster** | **1** | **2** | **3** | **1** | **2** | **3** |
| **HADDOCK score** | -51.9 ± 6.6 | -38.8± 7 | -27.3 ± 2.5 | -93.8 ± 2.6 | -54.7 ± 16.6 | -22.6 ± 8.2 |
| **Cluster Size** | 45 | 13 | 15 | 181 | 5 | 4 |
| **RMSD (Å)^#^** | 1.2 ±0.9 | 8.6 ± 0.2 | 10.8 ± 0.6 | 1.2 ± 0.8 | 10.7 ± 1.2 | 7.6 ± 1.3 |
| **Van de Waal energy (kcal/mol)** | -35 ± 11 | -27.7 ± 7.7 | -26.4 ± 4.2 | -50.0 ± 4.1 | -35.2 ± 7.5 | -16.8 ± 3.5 |
| **Electrostatic energy (kcal/mol)** | -258.1 ± 94.8 | -188.2 ± 31.9 | -186.6 ± 40.4 | -158.6 ± 33.8 | -42.7 ± 27.5 | -26.2 ± 25.4 |
| **Desolvation energy (kcal/mol)** | 33.7 ± 4.3 | 25.1 ± 6.7 | 33.4 ± 3.4 | -15.4 ± 2.8 | -18.8 ± 5.4 | -1.3 ± 2.0 |
| **Restrains Violations energy (kcal/mol)** | 10.5 ± 3.68 | 14.5 ± 12.92 | 29.9 ± 23.7 | 33.1 ± 22.93 | 78.2 ± 7.02 | 7.5 ± 1.78 |
| **Buried surface area (Å^2^)** | 1224.9 ± 95.3 | 1038.1 ± 134.1 | 1022.3 ± 63.3 | 1327.7 ± 54.3 | 1043.9 ± 173.8 | 700.6 ± 76.9 |

^#^ Root-Mean-Square-Deviation from the overall lowest energy structure
