# Supplementary material for: Structural Analysis of the UBA Domain of X-linked Inhibitor of Apoptosis Protein Reveals Different Surfaces for Ubiquitin-Binding and Self-Association
Source: PLoS One. 2011 Dec 15;6(12):e28511. doi: 10.1371/journal.pone.0028511 (PMC3240630; doi:10.1371/journal.pone.0028511)
Supplement: Methods S1 — Sample preparation of XIAP-UBA-RING and analytical size exclusion chromatography. (DOCX) [file pone.0028511.s007.docx]

**Sample preparation of XIAP-UBA-RING**

The DNA sequence encoding the XIAP-UBA-RING (Arg365-Ser497) was subcloned into pGB1-HIS bacterial expression vector. The expression and purification of ^15^N-labled XIAP-UBA-RING is essentially the same as described for that of XIAP-UBA. ^15^N-labled XIAP-UBA-RING was prepared in BisTris buffer (20 mM BisTris-HCl, pH 6.7, 150 mM NaCl, 5 mM d_10_-DTT, 1 mM PMSF, 90 % H_2_O/10 % D_2_O) for the NMR study.

**Analytical size exclusion chromatography**

The oligomerization state of XIAP-UBA-RING was investigated by analytical size exclusion chromatography using a Superdex 75 10/300 column (GE Healthcare). The column was pre-equilbrated with BisTris and XIAP-UBA-RING sample (protein concentration =0.18 mM) was applied at a flow rate of 0.5 ml/min. The elution profile was recorded and eluted fractions analyzed by SDS–PAGE. The column was calibrated using molecular mass standards (GE Healthcare). A calibration curve was plotted based on the elution volumes of these molecular mass standards. The apparent molecular mass for the XIAP-UBA-RING was then extrapolated from the calibration curve based on its elution volume.
